# Ossicular chain reconstruction in chronic otitis media: hearing results and analysis of prognostic factors^☆^

**DOI:** 10.1016/j.bjorl.2018.09.005

**Published:** 2018-10-18

**Authors:** Syriaco Atherino Kotzias, Mariana Manzoni Seerig, Maria Fernanda Piccoli Cardoso de Mello, Leticia Chueiri, Janaina Jacques, Martin Batista Coutinho da Silva, Daniel Buffon Zatt

**Affiliations:** Hospital Governador Celso Ramos, Departamento de Otorrinolaringologia, Florianópolis, SC, Brazil

**Keywords:** Chronic otitis media, Ossicular chain, Ossiculoplasty, Cartilage graft, Ossicular prosthesis, Otite média crônica, Cadeia ossicular, Ossiculoplastia, Enxerto de cartilagem, Prótese ossicular

## Abstract

**Introduction:**

The goal of ossiculoplasty is to improve hearing and the success of this procedure depends on several factors.

**Objective:**

Analyze the hearing results in patients with chronic otitis media undergoing ossicular chain reconstruction, as well as predictive factors for successful surgery.

**Methods:**

Charts of patients undergoing ossiculoplasty between 2006 and 2016 were reviewed. Sixty-eight patients were included, totaling 72 ears. The following data was analyzed: gender, age, smoking status, laterality, pathology, audiometric exams, type of surgery, previous surgery, characteristics of the middle ear, otorrhea and ossicular chain status. Patients were also classified according to two indices: middle ear risk index and ossiculoplasty outcome parameter staging. The results were evaluated by comparing the air-bone gap before and after surgery. The success of reconstruction was defined as air-bone gap ≤20 dB and the improvement of speech reception Thresholds, calculated through the mean frequencies 0.5, 1, 2 and 3 kHz.

**Results:**

Reconstruction success rate was 61%. The mean preoperative air bone gap was 34.63 dB and decreased to 17.26 dB after surgery. There was a correlation between low risk in middle ear risk index and ossiculoplasty outcome parameter staging indices with postoperative success. The most frequently eroded ossicle was the incus and the type of prosthesis most used was tragal cartilage. In the patients without incus, we achieved success in 74.2% of the surgeries. In the absence of the stapes, the success rate decreased to 63.3%. In the absence of the malleus, 85% of the patients had and air bone gap ≤20 dB.

**Conclusion:**

We achieved good audiometric outcomes in ossiculoplasty and the results are comparable to other centers. Ossicle status influenced postoperative results, especially in the presence of stapes. We also concluded that the indexes analyzed may help to predict the success of the surgery.

## Resumo

**Introdução:**

A ossiculoplastia tem como objetivo a melhoria da audição e o sucesso desse procedimento depende de diversos fatores.

**Objetivo:**

Analisar os resultados auditivos em pacientes com otite média crônica submetidos a reconstrução da cadeia ossicular, bem como os fatores preditivos de sucesso cirúrgico.

**Método:**

Prontuários de pacientes submetidos a ossiculoplastia entre 2006 e 2016 foram revistos. Sessenta e oito pacientes foram incluídos, total de 72 orelhas. Os seguintes dados foram analisados: sexo, idade, tabagismo, lateralidade, doença, exames audiométricos, tipo de cirurgia, cirurgia prévia, características da orelha média, otorreia e estado da cadeia ossicular. Os pacientes também foram classificados de acordo com dois índices: índice de risco da orelha média e estadiamento do parâmetro de desfecho da ossiculoplastia. Os resultados foram avaliados comparando o gap aéreo-ósseo antes e após a cirurgia. O sucesso da reconstrução foi definido como gap aéreo-ósseo ≤ 20 dB e a melhoria dos limiares de recepção de fala, calculados pelas frequências médias de 0,5, 1, 2 e 3 kHz.

**Resultados:**

A taxa de sucesso da reconstrução foi de 61%. O gap aéreo-ósseo pré-operatório médio foi de 34,63 dB e diminuiu para 17,26 dB após a cirurgia. Houve correlação entre baixo risco no índice de risco para orelha média e os índices de estadiamento do parâmetro de desfecho da ossiculoplastia com sucesso pós-operatório. O ossículo com erosão mais frequente foi a bigorna e o tipo de prótese mais utilizada foi a cartilagem tragal. Nos pacientes sem bigorna o sucesso foi alcançado em 74,2% das cirurgias. Na ausência do estribo, a taxa de sucesso diminuiu para 63,3%. Na ausência do martelo, 85% dos pacientes apresentaram gap aéreo-ósseo ≤ 20 dB.

**Conclusão:**

Melhora significativa da audição foi observada em pacientes submetidos à ossiculoplastia, os resultados foram comparáveis aos de outros centros. O “status” dos ossículos influenciou os resultados pós-operatórios, principalmente a presença do estribo. Também concluímos que os índices analisados podem ajudar a prever o sucesso da cirurgia.

## Introduction

Chronic Otitis Media (COM) is defined as; the presence of irreversible inflammatory disease in the ear cleft. It usually affects the ossicular chain leading to conductive hearing loss. Total or partial erosion of the ossicular chain is seen in about 80% of patients who present with Chronic Otitis Media with Cholesteatoma (COMC) whereas in the absence of cholesteatoma it can be present in approximately 20%. The incus is the ossicle most frequently affected, followed by the stapes and malleus.^1^

The objective of ossicular chain reconstruction is to restore the hearing impairment. There are many factors that can affect outcomes in this procedure including the middle ear environment, status of the eustachian tube, surgical technique, type of prosthesis and status of residual ossicular remnants.^2^, ^3^ In 1971, Austin classified ossicular chain disruption in 4 groups (A–D) based on the presence or absence of the malleus handle and the stapes arch. Kartush added three categories to this classification: intact ossicular chain (0), fixation of the malleus head (E) and fixation of the stapes (F).^4^ Kartush also described a score, named MERI (Middle Ear Risk Index), to stratify patients according to the severity of their disease. MERI score takes into consideration the presence or absence of otorrhea and cholesteatoma, tympanic membrane perforation, ossicular chain status (Austin–Kartush criteria), middle ear granulation and previous surgery (Table 1).^5^ In order to predict hearing outcomes in ossicular chain reconstruction, Dornhoffer and Gardner developed another index titled OOPS (Ossiculoplasty Outcome Parameter Staging). This index analyzes almost the same parameters as the MERI score does, however it classifies the ossicular chain as follows: normal or abnormal with or without malleus, not taking into account the status of stapes (Table 2).^2^, ^6^, ^7^

**Table 1:** Middle Ear Risk Index (MERI).

Risk factor	Risk value
*Otorrhoea*	
I Dry	0
II Occasionally wet	1
III Persistently wet	2
IV Wet, cleft palate	3

*Perforation*	
Absent	0
Present	1

*Cholesteatoma*	
Absent	0
Present	1

*Ossicular status (Austin/Kartush)*	
O: M+I+S+	0
A: M+S+	1
B: M+S−	2
C: M−S+	3
D: M−S−	4
E: Ossicle head fixation	2
F: Stapes fixation	3

*Middle ear granulation or effusion*	
No	0
Yes	1

*Previous surgery*	
None	0
Staged	1
Revision	2

MERI 0, normal; MERI 1–3, mild diseases; MERI 4–6, moderate disease; MERI 7–12, severe disease.

**Table 2:** Ossiculoplasty Outcome Parameter Staging (OOPS) index.

Rick factor	Risk value
*Middle ear factors*	
*Drainage*	
None	0
Present >50% of the time	1

*Mucosa*	
Normal	0
Fibrotic	2

*Ossicles*	
Normal	0
Abnormal, malleus+	1
Abnormal, malleus−	2

*Surgical factors*	
*Type of surgery*	
No mastoidectomy	0
Canal wall up masctoidectomy	1
Canal wall down mastoidectomy	2

*Revision surgery*	
No	0
Yes	2

The aim of this study is to verify the efficiency of these scores in predicting outcomes in ossiculoplasties, and also to evaluate the impact of the status of ossicular chain on the results.

## Methods

### Ethical committee approval

This study was approved by the ethics committee of our institution in July 4th, 2017 under protocol number 073627/2017.

### Patient selection

The records of all patients who underwent otological surgery by the senior author between February 2006 and December 2016 were reviewed. We included patients with chronic otitis media and ossicular chain erosion or disruption identified during the surgery in whom ossiculoplasty was performed. Congenital and tumor cases were excluded. We also excluded patients who did not have proper records of the surgery and pre- or postoperative audiograms. In patients requiring revision surgery, only the first ossiculoplasty performed by the senior author was included and if the surgery was staged, we included only the procedure in which ossiculoplasty was performed. However patients who had prior ossiculoplasty elsewhere and underwent revisional surgery were included. The following data was obtained from the reviewed charts: patient's sex, age at surgery, smoking status, COM with or without cholesteatoma, indication for surgery, laterality of procedure, procedure performed, history of previous surgery and if it was staged or revision, presence of preoperative otorrhea, intraoperative status of the mucosa and ossicles (presence or absence of incus, stapes and malleus). Ossicular chain reconstruction was performed using autologous ossicles, cortical bone or tragal cartilage. Patients were classified according to the OOPS and MERI index.

### Audiometric methods

Air-Conduction (AC) and Bone-Conduction (BC) thresholds at 0.5, 1, 2 and 3 kHz were recorded and used to calculate Pure-Tone Averages (PTAs). If the 3 kHz threshold was not recorded, the average of the 2 and 4 kHz was used. The PTA-ABG was the difference between AC PTA and BC PTA. Preoperative and postoperative Speech Reception Thresholds (SRTs) were recorded.

### Statistical analysis

Data was analyzed using IBM/SPSS 19 (Statistical Package of Social Science). Continuous variables were tested using Pearson's test. Comparisons of categorical variables were performed using the coefficient of Contingency. A value of *p* < 0.05 was considered indicative of statistical significance.

### Outcome measures

Primary outcome measures included hearing results and were measured from 90 days to 5 years after surgery. We considered a minimum follow-up period of 6 months. Successful ossiculoplasty was defined as a postoperative PTA-ABG ≤ 20 dB. The MERI and OOPS scores were determined for all patients. To compare the risk categories the patients were divided into six groups: for the OOPS index patients were classified as low risk (1–3), intermediate risk (4–6) and high risk (7–9), and according to the MERI score they were divided into mild disease (1–3), moderate disease (4–6) and severe disease (7–12). Then, for a detailed analysis, the type of surgery, middle ear disease, ossicular chain status according to Austin–Kartush classification presented in Table 1, the prostheses used were assessed, and the patients were divided into groups to compare the hearing results.

## Results

A total of 269 charts were reviewed between February 2006 and December 2016. Five patients were excluded because they did not have a diagnosis of chronic otitis media (three patients had temporal bone paragangliomas and two had congenital atresia of the ear) and 88 did not undergo ossiculoplasty. We also excluded 104 patients without proper records related to their diagnosis, surgery, clinical or audiometric data. In total, 68 patients were included in the study, totaling 72 ears.

**Figure 1:**
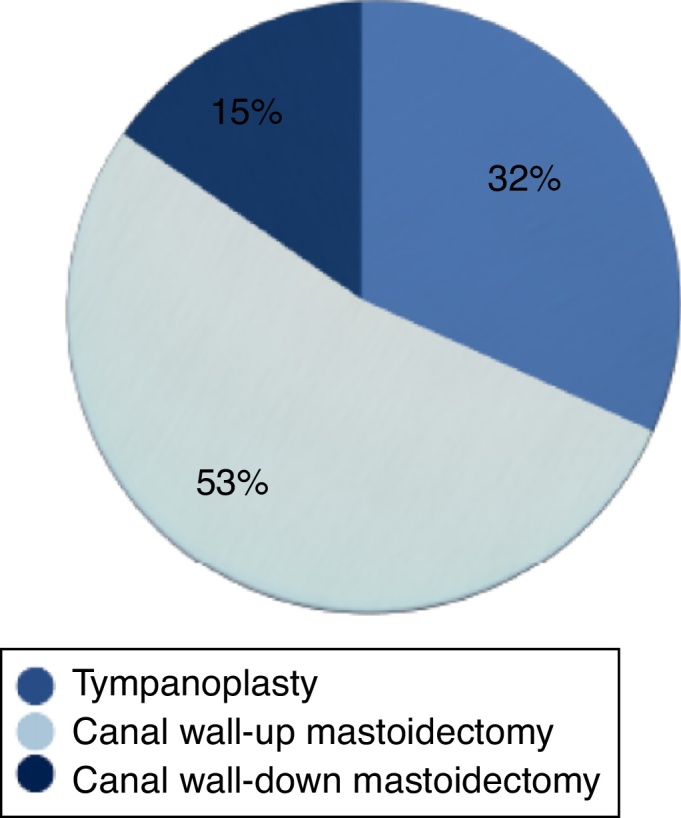
Type of surgery performed.

The mean age of patients was 36.6 ± 15.52: 54.1% were female and 48.61% were male. There was no prevalence of any ear (50% left or 50% right). The most common diagnosis was primary chronic otitis media with cholesteatoma (58.4%), followed by COM without cholesteatoma in 41.6% and in this last group 20.8% of the patients presented suppurative disease. Overall, otorrhea was found in 64% of the ears. Only 14% of the patients were smokers (Table 3). In total, 23 tympanoplasties, 38 Canal Wall-Up Mastoidectomies (CWUM) and 11 Canal Wall-Down Mastoidectomies (CWDM) were performed (Fig. 1).

**Table 3:** Demographic information.

Number of ears	72	
Mean age (years)	36.6 ± 15.52	
Sex, *n* (%)	Male	Female
	35 (48.6%)	37 (51.4%)
Laterality, *n* (%)	Right ear	Left ear
	36 (50%)	36 (50%)
Smoking status, *n* (%)	Yes	No
	10 (14%)	62 (86%)
Othorrea, *n* (%)	Yes	No
	46 (64%)	26 (36%)
Diagnosis, *n* (%)	Chronic non-suppurative otitis media	15 (20.8%)
	Chronic suppurative otitis media	15 (20.8%)
	Primary acquired cholesteatoma	40 (55.6%)
	Secondary acquired cholesteatoma	2 (2.8%)

**Figure 2:**
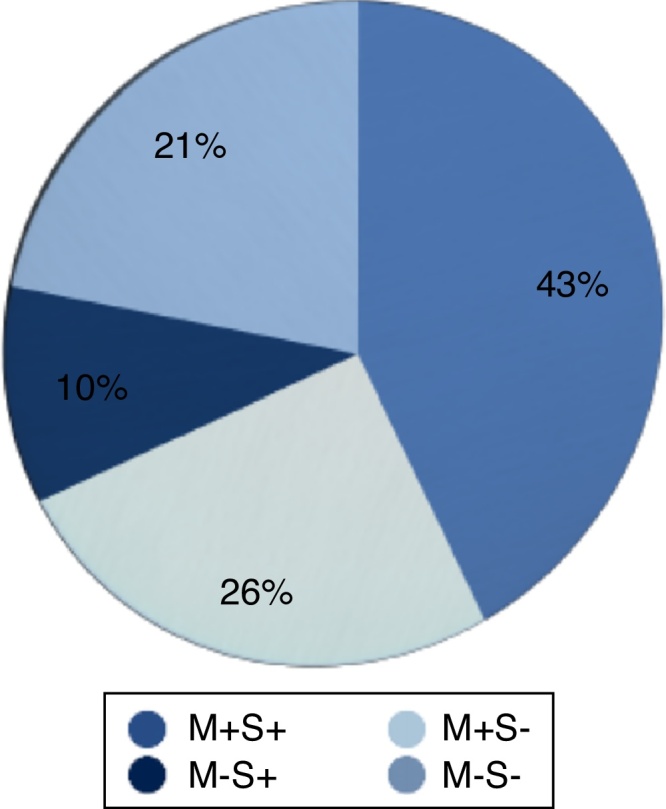
Pre-operative ossicular chain status. M+S+ indicates Malleus and Stapes present; M+S−, Malleus present and Stapes absent; M−S+, Malleus absent and Stapes present and M−S−, Malleus and Stapes absent.

Erosion of incus was the most frequent ossicular abnormality and it was seen in 31 (43%) of the ears, followed by erosion of stapes in 19 (26%) and erosion of malleus and stapes in 15 (21%). Malleus erosion with intact stapes was observed in only 7 ears (10%) (Fig. 2).

The mostly widely used prosthesis was tragal cartilage (*n* = 47) corresponding to 65% of the cases. Autologous incus was used in 17 (24%) patients and cortical bone was used in 8 (11%). The group of tragal cartilage presented a postoperative PTA-ABG ≤ 20 dB in 72.3% of the cases, while 59% of the patients of autologous incus and 62.5% of cortical bone presented a PTA-ABG > 20 dB (*p* = 0.04).

The average preoperative PTA-ABG was 34.63 ± 9.94 dB and postoperative PTA-ABG was 17.26 ± 12.92 dB. The mean preoperative SRT was 54.72 ± 14.98 dB and after surgery was 40.42 ± 20 dB, demonstrating an improvement of 14.30 dB (*p* < 0.05).

**Figure 3:**
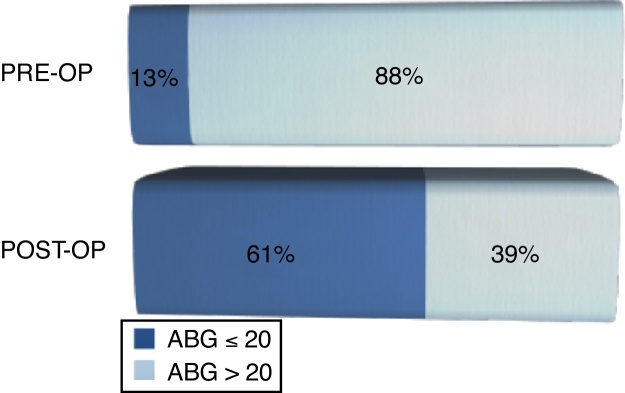
Comparison between pre-operative and post-operative ABG (Air Bone Gap).

Before surgery, 87.5% of the patients presented an ABG > 20 and 61% of the patients achieved a postoperative PTA-ABG ≤ 20 dB (Fig. 3). An improvement in SRT was observed in 77.8% after surgery, whereas 7% maintained the same SRT and 15.3% presented deteriorating results.

Three patients groups, based on low-risk (OOPS 1–3; Group 1), intermediate risk (OOPS 4–6; Group 2) and high risk (OOPS 7–9; Group 3) were arbitrarily created to evaluate the impact of the severity of the disease in the results of the ossicular chain reconstruction. Seventy three percent of the patients in group 1 achieved a closure of ABG to less than or equal to 20 dB, although this trend did not achieve statistical significance (*p* = 0.265) (Table 4).

**Table 4:** Correlation between OOPS Index and pre-operative Air Bone Gap (ABG).

OOPS	ABG ≤ 20 dB	ABG > 20 dB
Low risk (1–3)	73.1% (19)	26.9% (7)
Intermediate risk (4–6)	53.5% (23)	46.5% (20)
High risk (7–9)	66.7% (2)	33.3% (1)
Total	61.1% (44)	38.9% (28)

*p* > 0.05.

Patients were also divided into three groups according to the MERI index: mild disease (MERI 1–3; Group 4), moderate disease (MERI 4–6; Group 5) and severe disease (MERI 7–12; Group 6). We observed that 70% of patients in Group 4 presented an ABG ≤ 20 dB at follow-up, while in Group 6, 64% presented an ABG > 20 dB, demonstrating that patients with mild disease according to the MERI index had better postoperative results than patients with severe disease (*p* = 0.01) (Table 5).

**Table 5:** Correlation between MERI index and pre-operative Air Bone Gap (ABG).

MERI index	ABG ≤ 20 dB	ABG > 20 dB
Mild disease (1–3)	70% (7)	30% (3)
Moderate disease (4–6)	72.5% (29)	27.5% (11)
Severe disease (7–12)	36.4% (8)	63.3% (14)
Total	61.1% (44)	38.9% (28)

*p* < 0.05.

In regard to the ossicular chain, we found that the ossicle that was the most frequently eroded was the incus (*n* = 31). The best results of ossicular chain reconstruction were achieved in Austin–Kartush Group A (74% of the patients presenting an ABG ≤ 20 dB), followed by Group C (85.7% achieving an ABG ≤ 20 dB), and the worst results were found in patients with stapes erosion (ABG ≤ 20 dB in 63.2% of the patients) and patients without malleus and stapes (80% of them present an ABG > 20 dB) (*p* = 0.02) (Table 6).

**Table 6:** Correlation between Austin–Kartush criteria and hearing results according to post-operative Air Bone Gap (ABG).

	ABG ≤ 20 dB	ABG > 20 dB	Total
M+S+	23 (74.2%)	8 (25.8%)	31 (100%)
M+S−	12 (63.2%)	7 (36.8%)	19 (100%)
M−S+	6 (85.7%)	1 (14.3%)	7 (100%)
M−S−	3 (20%)	12 (80%)	15 (100%)

Total	44 (61%)	28 (39%)	72 (100%)

M+S+, Malleus and stapes present; M+S−, Malleus present and stapes absent; M−S+; Malleus absent and stapes present; M−S−, Malleus and stapes absent.

*p* < 0.05.

## Discussion

In patients with COM the objective of the surgery is to provide a dry ear, ensure the function of Eustachian tube, and restore the sound-conducting system of the middle ear. Ossicular chain reconstruction represents a challenge even for experienced otologists and the success is achieved with good and long-lasting audiometric outcome, represented by closure of postoperative ABG less or equal do 20 dB.^2^, ^5^ In our study, 61% of the patients presented with an ABG ≤ 20 dB, and this result was similar to other series.^2^, ^8^, ^9^, ^10^, ^11^ We also observed an improvement of a mean 14.3 dB in postoperative speech reception thresholds. The mean ABG before surgery was 34.63 dB and decreased to 17.26 dB after reconstruction, showing an improvement of 17.36 dB.

The ideal reconstruction should be easy to perform and the materials should be well tolerated by the patient, with low reabsorption or extrusion rates, and it should provide good hearing results over the time. A wide variety of materials can be used for ossicular reconstruction, i.e. autografts (autologous incus, tragal cartilage, cortical bone), bone cement or a prosthesis. We believe that autograft materials are a good alternative because of the biocompatibility, low extrusion rate, and their lower price compared with other groups of prosthesis. Emir et al., reported 58.1% of success using autologous incus and 71.4% using cortical bone.^11^ O’Connel et al., described a series of 156 patients who underwent ossiculoplasty with titanium prosthesis, and they found 67% of the patients achieving ABG ≤ 20 dB in short-term follow-up (<6 months).^12^ In this series, the majority of the reconstructions were with tragal cartilage, achieving 72.3% of success and with a mean follow-up (including audiometric exams) of one year. We generally use tragal cartilage in the presence of the stapes, placing it between the capitulum and handle of malleus. If the stapes is absent or eroded we use cortical bone, placing it from the footplate to the handle of the malleus, or to the tmpanic membrane if malleus is absent. We suggest that the absence of stapes probably explains our poor results with cortical bone.

The use of a risk stratification index allows surgeons to compare patients according to preoperative conditions, can help to predict hearing results and risks for complications or recurrence, and it also can be useful for patients, providing them useful information about their disease, so they can modify risk factors related to the failure of the surgery. However, the creation of an ideal tool is difficult because of variations in different populations, surgeon's skills, surgical techniques, and elements extrinsic to the middle ear (smoking habits, adenoidal disease, living environment and gastroesophageal reflux).^6^ Dornhoffer and Gardner created the OOPS index, considering that the absence of malleus, fibrotic middle ear mucosa, and otorrhea could be useful in prognosticating hearing outcomes after ossiculoplasties.^7^ Cox et al. identified a strong positive correlation between OOPS index score and average post-operative PTA-ABG and they found that hearing results tend to remain stable, especially in lower-risk patients.^2^ In our study we demonstrated better audiometric outcomes in Group 1 patients (73.1% achieved a PTA-ABG ≤ 20 dB) but it was not statistically significant. When using this index we found more than half of the patients classified as intermediate risk, and only three patients in the high-risk group.

MERI index, described by Kartush, stratifies patients according to the environment of the middle ear (presence of otorrhea, granulation) perforation of tympanic membrane, existence of cholesteatoma and it gives a different score for each kind of ossicular erosion or fixation.^11^ In the present study, 70% of patients classified as mild disease (score 1–3) presented a post-operative ABG closure ≤20 dB, while in the group of severe disease (score 7–12) we achieved success only in 36.4% of the patients (*p* = 0.017).

When it comes to the damage of individual ossicles as a determinant of hearing results in ossiculoplasties, the literature data is controversial. Many authors believe that the presence of the malleus predicts the best outcomes regardless of the condition of stapes.^4^, ^7^, ^13^, ^14^ Blom et al. analyzed Austin–Kartush groups in their meta-analysis and demonstrated a significant difference in mean ABG in favor of Group B (11.1) when compared to Group C (15.7).^4^ Bared and Angeli reported presence of malleus handle as a favorable prognostic factor on ossicular chain reconstruction.^14^ It is known that the malleus contributes to the stability of the tympanic membrane and consequent stability of the ossicular chain. It also prevents graft lateralization and acts to improve sound conduction through the catenary lever.

On the other hand, there are some authors who consider that the presence of stapes superstructure is crucial in audiometric outcomes. Castro et al. reviewed 153 ossiculoplasties and found that the mean postoperative ABG in patients with stapes superstructure was 11.6 ± 10.2 dB, while in patients without stapes this mean increased to 17.7 ± 9.91, associating the success rate to the presence of this ossicle.^3^ In our study, we observed that in patients with a missing or eroded incus (Austin–Kartush Group A) the interposition was successful in 74.2%. Comparing patients in Group B and C, we achieved better results in Group C, in which the stapes supraestructure was present, with 85.7% of success. Patients in Group B presented ABG ≤ 20 dB in 63% of the cases and patients in Group D had the worst results (80% remained with ABG > 20 dB). Therefore, based on this series, we can observe that the presence of stapes superstructure is a good predictor for success in ossicular chain reconstruction.

## Conclusion

In the present study we have classified our patients according to two classification systems and concluded that the OOPS index seems not to be accurate on prognosticating hearing outcomes, while the MERI index can be a valuable tool for surgeons to estimate the risks, predict success of the surgery as well as to select the best candidates for reconstruction. Regarding the ossicular chain we concluded that the status of each ossicle is important for surgical success, especially the presence of stapes. This series surgical success was 61% of the cases and it also showed an improvement of 17.26 in PTA-ABG after surgery. In the presence of stapes superstructure the success rate was 85.7%.

## Conflicts of interest

The authors declare no conflicts of interest.
